# Solamargine Alleviated UVB-Induced Inflammation and Melanogenesis in Human Keratinocytes and Melanocytes *via* the p38 MAPK Signaling Pathway, a Promising Agent for Post-inflammatory Hyperpigmentation

**DOI:** 10.3389/fmed.2022.812653

**Published:** 2022-06-13

**Authors:** Juemin Zhao, Yanjun Dan, Ziqi Liu, Qianqian Wang, Min Jiang, Chengfeng Zhang, Hamm-Ming Sheu, Chrang-Shi Lin, Leihong Xiang

**Affiliations:** ^1^Department of Dermatology, Huashan Hospital, Fudan University, Shanghai, China; ^2^Kao Chao-Hsing Dermatologic Clinic, Kaohsiung City, Taiwan; ^3^Department of Dermatology and Family Medicine, National Yang-Ming Chiao-Tung University, Taipei City, Taiwan; ^4^Dr. Lin Skin Clinic, Taipei City, Taiwan

**Keywords:** post-inflammatory hyperpigmentation (PIH), SM (solamargine), p38, MAPK, Nrf2, HO-1, inflammation, melanogenesis

## Abstract

Post-inflammatory hyperpigmentation (PIH) is a common acquired pigmentary disorder occurring after skin inflammation or injury. Ultraviolet B irradiation could exaggerate PIH clinically due to its effect on promoting cutaneous inflammation and melanogenesis in keratinocytes and melanocytes, respectively. Solamargine (SM), a steroidal alkaloid glycoside extracted from *Solanum undatum*, significantly inhibits Ultraviolet B (UVB)-induced pro-inflammatory cytokines IL-1α, IL-1β, IL-8, and IFN-γ, as well as paracrine melanogenic factors ET-1, α-MSH, and bFGF in human keratinocytes. Additionally, SM significantly attenuated UVB-induced melanin synthesis in human epidermal melanocytes through down-regulation of tyrosinase activity and expression of MITF, TRP-1, TRP-2, and tyrosinase. SM exerted an anti-inflammatory effect in UVB-irradiated keratinocytes through the p38 MAPK/Nrf2/HO-1 signaling pathway. With its anti-inflammatory and whitening effect, SM may improve PIH through paracrine regulations of keratinocytes and direct action on melanocytes, making it a promising agent for PIH.

## Introduction

Post-inflammatory hyperpigmentation (PIH) is an acquired hypermelanosis of the epidermis or dermis following cutaneous inflammation or injury. Although clinicians observed pigmentary change in all skin types, it more frequently affects individuals with Fitzpatrick skin types (FST) IV-VI due to the increased reactivity of melanocytes within the skin (1, 2). The treatment of PIH remains intractable. Except for a few anti-inflammatory agents, current therapies mainly target the pigmentary phase, which is primarily unsatisfactory. Current treatments demonstrate inconsistent efficacies and different disadvantages: (1) depigmentation agents (e.g., hydroquinone, azelaic acid, tretinoin, and 4-hydroxyanisole) which are prone to irritant reactions and hypopigmentation in clinical use (3); (2) retinoids (e.g., retinoic acid, adapalene, tazarotene, etc.) which could cause irritant dermatitis such as redness, desquamation, and pruritus (4); (3) glucocorticoids (e.g., betamethasone, desonide, hydrocortisone butyrate, etc.) which could lead to adverse reactions such as skin thinning, atrophy, and capillary dilation with long-term use, even cause hyperpigmentation in some cases (3); (4) chemical peeling (e.g., glycolic acid, salicylic acid, trichloroacetic acid, etc.) which may cause burning and irritating during operation, thus increasing the risk of hyperpigmentation (5, 6); (5) laser therapy (e.g., intense pulsed light and 532, 694, and 1064 nm Q-switched lasers) which are expensive and may exaggerate hyperpigmentation (7–9). In addition to the adverse effects of the above treatments, improper timing of interventions may induce new inflammatory stimuli to result in aggravation of hyperpigmentation. It is of great interest for clinicians to provide targeted anti-inflammatory or whitening therapy depending on the stage of PIH. As our team previously reported, inflammatory and pigmentary phases were overlapped rather than isolated, so drugs with both anti-inflammatory and anti-pigmentary effects are ideal options for treating PIH (10). Plant extracts such as arbutin, licorice extract, soybean extract, and green tea polyphenols (11) have become popular choices in PIH patients due to their mild effects and high tolerance. New natural extracts with more potent anti-inflammatory and whitening effects are still of great clinical interest.

**FIGURE 1:**
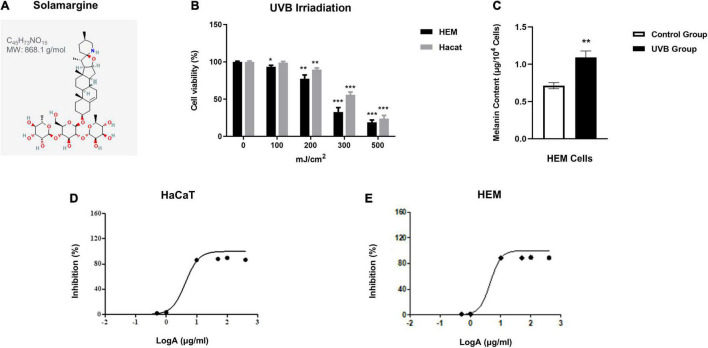
Cell viability and melanin content of HaCaT and HEM cells after NB-UVB irradiation. The inhibition rate of HaCaT cells and HEM cells of different concentrations of SM. **(A)** The chemical structure of SM modified from https://pubchem.ncbi.nlm.nih.gov/compound/Solamargine. **(B)** Cell viability of HaCaT cells and HEM cells was detected by CCK-8 24 h after irradiation of NB-UVB with different fluences (100, 200, 300, and 500 mJ/cm^2^). Cell viability is presented as a eprcentage of control. **(C)** Melanin content of HEM cells after irradiation of NB-UVB with 250 mJ/cm^2^ Melanin content is percentage as a percentage of control. **(D,E)** The IC50 value for HaCaT cells was 4.252 μg/ml and for HEM cells was 4.472 μg/ml. *P < 0.05, **P < 0.01, and ***P < 0.001.

Solamargine (SM), a steroidal alkaloid glycoside extracted from *Solanum undatum*, has been proven to have anti-inflammatory, antioxidant and anti-tumor effects. Studies have shown that SM can exert anti-tumor effects (Figure 1A) by up-regulating the expression of TNF-α1 and TNF-α2 and activating caspase-3 to cause tumor cells apoptosis (12, 13); it can exert potent anti-inflammatory effects by inhibiting the NF-κB/p65 pathway and inhibiting the expression of COX-2 (14, 15). In addition, SM can exert some antioxidant effects by reducing ROS release and down-regulating the activity of i-NOS (16). SR-T100 solution, a novel water-soluble product whose active ingredient is SM at a concentration of 5.53 mg/ml (purity >99%), is currently used in Taiwan, China, for the treatment of solar keratosis, condyloma acuminata and viral warts with particular efficacy (17–22).

Since inflammation and oxidative stress contributed to PIH, and SM has anti-inflammatory and antioxidant effects, we speculate whether it could have therapeutic effects on PIH through the dual effects of anti-inflammation and anti-pigmentation. Current laboratory studies of SM have focused on the antitumor effects against tumor cells, and few studies reported the effects on keratinocytes and melanocytes. We examined the effects of SM in the UVB-induced PIH model on pro-inflammatory cytokines and melanogenic factors in keratinocytes and melanin synthesis in melanocytes after UVB irradiation and further investigated possible signaling pathways. We hope that studies on this novel compound’s biological functions and mechanisms could provide a basis for screening future novel drugs and lay the theoretical foundation for clinical translation.

## Materials and Methods

### Cell Culture and Ultraviolet B Irradiation

Human keratinocyte cells and Human Epidermal Melanocyte (HEM) cells were cultured at 37°C in a humidified CO_2_ incubator (95% air, 5% CO_2_) in Dulbecco’s modified Eagle’s medium (Gibco, United States) supplemented with 10% fetal bovine serum and 1% penicillin-streptomycin. After washing twice with phosphate-buffered saline (PBS), cells were irradiated with a thin cover of PBS, using handheld NB-UVB light (wavelength of 311 nm, SH1B, Sigma, Shanghai, China). The dose rate was 12 mW/cm2, and the test dose ladder was 100, 200, 300, 400, and 500 mJ/cm^2^, with radiation times corresponding to 8, 17, 25, 33, and 42 s, respectively. Non-irradiated cells were covered with foil to prevent exposure. After UVB irradiation, PBS was removed and cells were incubated in fresh medium with or without SM for an additional 24 or 48 h before further experiments.

### Assessment of Viability

Human keratinocyte cells and HEM cells (2 × 10^3^ per well) were collected and seeded in triplicate 96-well plates in 100 μl of growth medium. Different seeding densities were optimized at the beginning of the experiments. 24 h after irradiation or SM treatment, Cell Count Kit-8 assay (Dojindo Laboratories, Kumamoto, Japan) was used to assess the cell viability of the HaCaT cells and HEM cells according to the manufacturer’s instructions. Next, the absorbance of each well was measured at an absorbance of 475 nm with the Spectra Max 384 PLUS microplate reader (Molecular Devices, Sunnyvale, CA, United States).

### Melanin Content Assay

HEM cells were seeded in a 60 mm culture dish at a density of 5 × 10^5^ cells per well. 24 h after UVB irradiation or SM treatment, HEM cells were dissolved in 500 μL of 1 m NaOH with 10% DMSO for 1 h at 80°C and the relative melanin content was measured at an absorbance of 405 nm with the Spectra Max 384 PLUS microplate reader (Molecular Devices, Sunnyvale, CA, United States).

### Tyrosinase Activity Assay

HEM cells were seeded in a 60 mm culture dish at a density of 5 × 10^5^ cells per well. 24 h after UVB irradiation or SM treatment, HEM cells were washed with PBS and then were solubilized with 1% Triton X-100 at room temperature for 15 min. The lysates were centrifuged at 12,000 rpm for 10 min to obtain a supernatant. After quantification, 100 μl of lysis buffer was transferred into the 96-well plate with 100 μl of 1 mM L-DOPA added to each well. After incubation at 37°C for 1 h, absorbance was measured at 490 nm with the Spectra Max 384 PLUS microplate reader (Molecular Devices, Sunnyvale, CA, United States).

### Reverse-Transcription PCR

Total RNA was prepared from the treated cells using Trizol (Life Technologies, Rockville, MD, United States). The quality and quantity of isolated RNA samples were assessed by measuring the 260/280 nm absorption ratio of RNA. Extracted RNA was used to generate cDNA using PrimeScript RT Master Mix Kit (Takara, Japan). The cDNA was applied as a template for quantitative RT-PCR, which was carried out in a Real-Time PCR 7500 System (Applied Biosystems, Invitrogen) with SYBR Premix Extaq (Takara, Japan) in a 40-cycle PCR. The denaturing, annealing and extension conditions of each PCR cycle were 95°C for 30 s, 95°C for 5 s, and 60°C for 34 s, respectively. The primers used were as follows: *IL-1*α, 5′-GAGGGAGTCATTTCATTGGCG-3′, 5′ ATGCAGCCTTCATGGAGTGG -3′, *IL-1*β, 5′- CTGAGCT CGCCAGTGAAATG -3′, 5′- TGTCCATGGCCACAACAACT -3′, *IL-8*, 5′- CAGTGCATAAAGACATACTCCAAACC -3′, 5′- TGGTCCACTCTCAATCACTCTCA -3′, *IFN-*γ, 5′- GAATGT CCAACGCAAAGCAA -3′, 5′- GCTGCTGGCGACCGTT -3′, *MITF*, 5′- TGGTTTGGGCTTGTTGTTTGT -3′, 5′- CTGC ACCCGGGAATCG -3′, *TYR*, 5′- GCTTGTGAGCTTGCT GTGTC -3′, 5′- GGTCAGGCTTTTTGGCCCTA -3′, *TRP-1*, 5′- AAGCTTTTCTTGCTGGCGTG -3′, 5′- CTCCCA AGCACATCTACGCA -3′, *TRP-2*, 5′- TTCATCTGCTCATATCT GGTTTCTG -3′, 5′- CAGACAAGCCACGCCCTAGA -3′, *ET-1*, 5′- CCCTCCAGAGAGCGTTATGTG -3′, 5′- CCCGAAGGT CTGTCACCAA -3′, α*-MSH*, 5′- CCCCTGGTGACGCTGTTC -3′, 5′- CCCTCACTCGCCCTTCTTG -3′, *bFGF*, 5′- TGGATA GTGTGAGAGAATTAGGCTGTA -3′, 5′- TTTTGTAGTGGT GTGAATGCTGAA -3′, *GAPDH*, 5′-GCACCGTCAA GGCTGAGAAC-3′, 5′-ATGGTGGTGAAGACGCCAGT-3′. The relative expression levels were calculated with ΔΔCt method. The mRNA levels of each target gene were normalized to the levels of GAPDH.

### Western Blot Analysis

Human keratinocyte cells and HEM cells incubated under the indicated conditions were lysed, and total protein was extracted from HEM cells by the RIPA buffer, separated by 10% SDS-PAGE, and electro- transferred onto a polyvinylidene difluoride membrane (Millipore, Billerica, MA, United States). After blocking non-specific antibody binding by incubating the membrane in Tris-buffered saline containing 0.1% Tween-20 and 5% non-fat dried milk for 2 h at room temperature, the membranes were incubated at 4°C overnight with the following primary antibodies: from Abcam (Cambridge, MA, United States): anti-MITF (ab59232; 1:1000), anti-TYR (ab61294; 1:1000), anti-TRP-1 (ab83774; 1:500), TRP-2 (ab221144; 1:500), Nrf-2 (ab62352; 1:1000), HO-1 (ab52947; 1:2000), and from Cell Signaling Technology (Boston, MA, United States): p-p38 (ab178867; 1:1000), p38 (ab182453; 1:1000) and from Proteintech (Rosemont, United States):α-Tubulin (ab7291;1:10000). p38 inhibitor (SB203580) was purchased at Selleck and arbutin was purchased at Sigma. Afterward, the membranes were incubated with the appropriate horseradish peroxidase (HRP)-conjugated secondary antibody for 1 h at room temperature. Immunoreactive bands were detected by the Enhanced Chemiluminescence substrate (Pierce, Rockford, IL, United States) and visualized on the LAS-3000 Luminescence Image Analyzer (Fujifilm, Tokyo, Japan). Densitometric analysis was performed using the ImageJ software program.

### ELISA Assay

The culture supernatants of the treated HaCaT cells were collected 48 h after irradiation and IL-1α, IL-1β, IL-8, IFN-γ, ET-1, α-MSH, bFGF level was detected using ELISA Kits from Abcam (Cambridge, MA, United States) following the manufacturer’s instructions. Quantitative data analysis was performed using Raybio Q Analyzer software (RayBiotech, Inc.).

### Statistical Analysis

Data are expressed as mean ± S.D. The differences among the three groups were analyzed using one-way analysis of variance (ANOVA) followed by the least square difference (LSD) *post-hoc* test. *P* < 0.05 was considered statistically significant. Statistical tests were performed using SPSS software (version 21.0, Chicago, IL, United States). Graphical representations of the data were performed by GraphPad Prism (GraphPad Software Inc, San Diego, CA, United States).

## Results

### Ultraviolet B-Induced Inflammation and Hyperpigmentation in Keratinocytes and Melanocytes as an *in vitro* Model for Post-inflammatory Hyperpigmentation

According to previous studies, the doses of NB-UVB treatment of human keratinocytes and melanocytes generally vary from 30 to 300 mJ/cm^2^ (23–25). This study used 100, 200, 300, and 500 mJ/cm^2^ UVB to irradiate HaCaT cells and HEM cells, respectively. The cell morphology was observed by phase-contrast microscopy 24 h after irradiation, and the cell viability was measured by CCK-8. The results showed that with increasing irradiation dosage, cell density decreased, morphology was irregular, and cell proliferation viability was inhibited correspondingly.

After irradiating HaCaT cells and HEM cells at a dose of 300 mJ/cm^2^ UVB, the cell density decreased, the morphology was less regular, and the cell proliferation viability was significantly decreased (Figure 1B). The melanin content increased significantly after irradiating HEM cells using 250 mJ/cm^2^ UVB (Figure 1C). Therefore, 250 mJ/cm^2^ was chosen to establish the PIH model as the irradiation dose.

### The Effect of Solamargine on Cell Viability of Human Keratinocyte Cells and HEM Cells

Previous literature has shown that SM intervenes in tumor cell lines and normal cell lines with different IC50 values for different cells, with safe doses within 10 μg/ml (26).

The results showed that SM at 0.1 μg/ml had a mild pro-proliferative effect on HaCaT cells and HEM cells, and SM at 10 μg/ml and above had a strong inhibitory effect on both cells (Table 1). The IC50 value for HaCaT cells was 4.252 μg/ml and for HEM cells was 4.472 μg/ml. The log curves for different concentrations of SM are shown in Figures 1D,E. This study selected 1 and 2 μg/ml of SM as the intervention concentrations for HaCaT cells and HEM cells, respectively.

**TABLE 1:** Cell viability of HaCaT cells and HEM cells at different concentrations of SM.

SM concentration (μg/ml)	HaCaT cell inhibition rate (%)	HEM cell inhibition rate (%)
0	0 ± 1.01	0 ± 0.84
0.1	−2.91 ± 0.88	−2.79 ± 0.89
0.5	1.91 ± 1.93	1.22 ± 0.95
1	3.54 ± 1.20	1.21 ± 1.27
10	86.08 ± 1.09	88.34 ± 0.35
50	87.90 ± 0.50	88.21 ± 0.10
100	89.48 ± 0.33	89.36 ± 0.19
400	86.41 ± 4.33	88.88 ± 016

### Solamargine Attenuated UVB-Induced Secretion of Pro-Inflammatory Cytokines (IL-1α, IL-1β, IL-8, IFN-γ) and Melanogenic Factors (ET-1, α-MSH, bFGF) in Human Keratinocyte Cells

To evaluate the anti-inflammatory and anti-pigmentation ability of SM in UVB-irradiated HaCaT cells, we detected mRNA and protein expression of pro-inflammatory cytokines (IL-1α, IL-1β, IL-8, IFN-γ) and paracrine melanogenic factors (ET-1, α-MSH, bFGF) in HaCaT cells.

**FIGURE 2:**
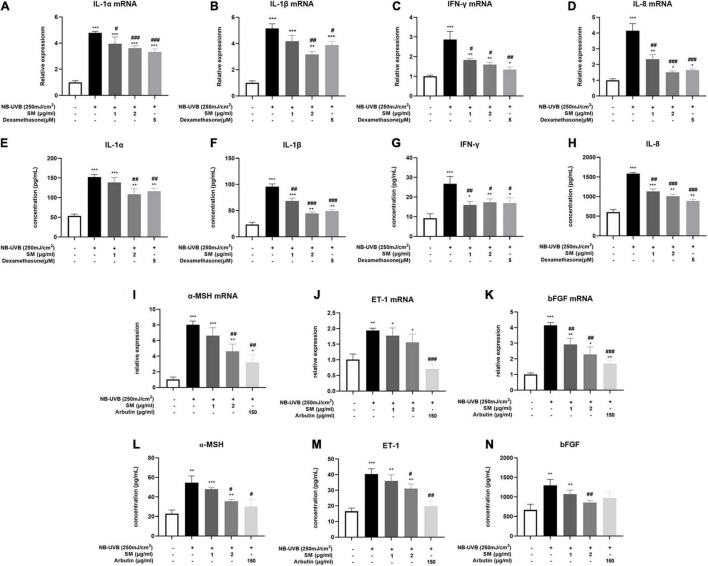
Solamargine (SM) attenuated UVB-induced secretion of pro-inflammatory cytokines and melanogenic factors in HaCaT cells. Real-time PCR (24 h after incubation with SM) and ELISA assay (48 h after incubation with SM) demonstrated SM attenuated UVB-induced secretion of pro-inflammatory cytokines, IL-1α **(A,E)**, IL-1β **(B,F)**, IL-8 **(C,G)**, and IFN-γ **(D,H)** in HaCaT cells. Treatment with SM after UVB irradiation significantly decreased the mRNA and protein levels of IL-1α, IL-1β, IL-8, and IFN-γ. The anti-inflammatory effect of SM is comparable to 150 μg/ml dexamethasone. Real-time PCR and ELISA assay demonstrated SM attenuated UVB-induced secretion of melanogenic factors, ET-1 **(I,L)**, α-MSH **(J,M)**, and bFGF **(K,N)** in HaCaT cells. Treatment with SM after UVB irradiation significantly decreased the mRNA and protein levels of ET-1, α-MSH, and bFGF. The anti-melanogenic effect of SM is comparable to 150 μg/ml Arbutin. The data were represented as mean ± SD from three independent experiments. *P < 0.05, **P < 0.01, ***P < 0.001 (vs. non-treated group); ^#^*P* < 0.05, ^##^*P* < 0.01; ^###^*P* < 0.001 (vs. NB-UVB irradiated group).

The results showed that the mRNA and protein expression of IL-1α, IL-1β, IL-8, and IFN-γ were significantly increased after UVB irradiation of HaCaT cells. SM at 1 and 2 μg/ml significantly inhibited the mRNA expression of IL-1α, IL-8, and IFN-γ in HaCaT cells after UVB irradiation. Upon treatment of 1 and 2 μg/ml SM, the mRNA and protein levels of IL-1α, IL-1β, IL-8, and IFN-γ significantly reduced compared to UVB irradiation alone, the anti-inflammatory effect of which is comparable to 150 μg/ml dexamethasone (Figures 2A–D).

In addition to pro-inflammatory cytokines, previous studies reported that certain paracrine factors secreted by keratinocytes, including ET-1, α-MSH, and bFGF (27, 28), could bind directly to corresponding receptors on melanocytes and promote melanogenesis upon inflammatory stimulation and UVB irradiation. The mRNA expression of the ET-1, α-MSH, and bFGF were all significantly increased after UVB irradiation of HaCaT cells (Figures 2I–K). There were also significantly higher protein concentrations in the cell supernatant of ET-1, α-MSH, and bFGF after UVB irradiation (Figures 2L–N). Similarly, at both mRNA and protein levels, 2 μg/ml SM significantly inhibited UVB-induced secretion of ET-1, α-MSH, and bFGF in HaCaT cells (Figures 2E–H). 150 μg/ml Arbutin was chosen to be a positive control. These results indicated SM prevented both pro-inflammatory and pro-melanogenic effects in UVB-irradiated HaCaT cells, making it a promising therapeutic alternative for the inflammatory phase of PIH.

### Solamargine Inhibited UVB-Induced Melanogenesis in Human Epidermal Melanocyte Cells

**FIGURE 3:**
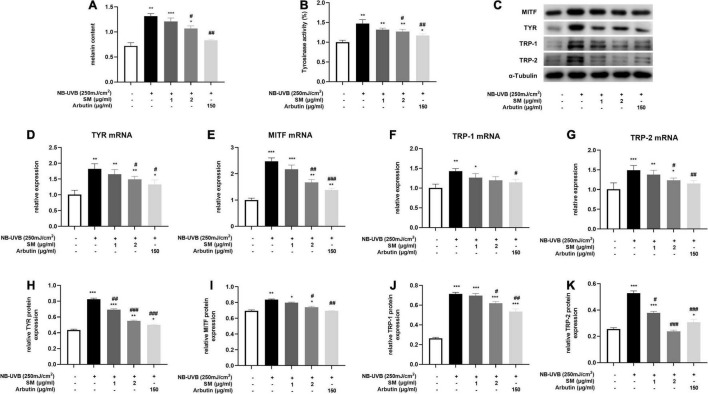
Solamargine (SM) Inhibited UVB-induced melanogenesis in HEM cells. **(A)** Melanin content of HEM cells 48 h after the UVB irradiation with SM or arbutin treatment. **(B)** Tyrosinase activity of HEMs 24 h after the UVB irradiation with SM or arbutin treatment. Tyrosinase activity is presented as a percentage of control. Real-time PCR (24 h after incubation with SM) and Western Blot (48 h after incubation with SM) demonstrated SM attenuated UVB-induced secretion of melanogenic factors, TYR **(C)** Western blot assay of MITF, TYR,TRP-1 and TRP-2 after UVB exposure with/without SM or arbutin treatment. **(D,H)**, MITF **(E,I)**, TRP-1 **(F,J)**, and TRP-2 **(G,K)** in HEM cells. Treatment with SM after UVB irradiation significantly decreased the mRNA and protein levels of TYR, MITF, TRP-1, and TRP-2. The anti-melanogenesis effect of SM is comparable to 150 μg/ml Arbutin. The data represent the mean ± S.D. from three independent experiments. *P < 0.05, **P < 0.01, ***P < 0.001 (vs. non-treated group); ^#^*P* < 0.05, ^##^*P* < 0.01, ^###^*P* < 0.001 (vs. NB-UVB irradiated group).

Melanin contents are closely related to melanosome maturation. Microphthalmia-associated transcription factor (MITF) is the central transcriptional factor governing the expression of melanogenic enzymes such as tyrosinase-related protein (TRP)-1, TRP-2 and tyrosinase (TYR) (29). To evaluate the role of SM in melanogenesis, we examined the melanin contents, tyrosinase activity, and the expression of several critical melanogenic factors, such as MITF, TRP-1, TRP-2, and TYR on both mRNA and protein levels. HEM cells were irradiated with 250 mJ/cm^2^ UVB and incubated with 1 μg/ml, 2 μg/ml of SM and 150 μg/ml of arbutin (a classical whitening component as a positive control) for 24 h. The results showed a significant increase in melanin contents (Figure 3A) and tyrosinase activity (Figure 3B) in the UVB group compared to the negative control group, and a slight decrease in melanin content in the UVB + 1 μg/ml SM group compared to the UVB group, but the difference was not statistically significant. Melanin contents and tyrosinase activity in the UVB + 2 μg/ml group and UVB + arbutin group decreased significantly compared to the UVB irradiated group.

Microphthalmia-associated transcription factor, TRP-1, TRP-2, and TYR mRNA and protein levels were significantly increased after UVB irradiation in HEM cells. SM at 2 μg/ml significantly inhibited the mRNA expression of TYR, MITF, and TRP-2 in HEM cells after UVB irradiation and to some extent on TRP-1 mRNA expression, but there were no statistically significant differences (Figures 3D–G). At the protein level, SM at 2 μg/ml significantly inhibited the expression of TYR, MITF, TRP-1, and TRP-2 proteins in HEM cells after UVB irradiation (Figures 3C,H–K). These results indicated that 2 μg/ml of SM could significantly inhibit the expression of critical regulatory molecules of melanin synthesis in HEM cells after UVB irradiation, comparable to 150 μg/ml arbutin.

### The Effect of Solamargine Exerted Through p38 MAPK/Nrf2/HO-1 Pathway in Human Keratinocyte Cells

**FIGURE 4:**
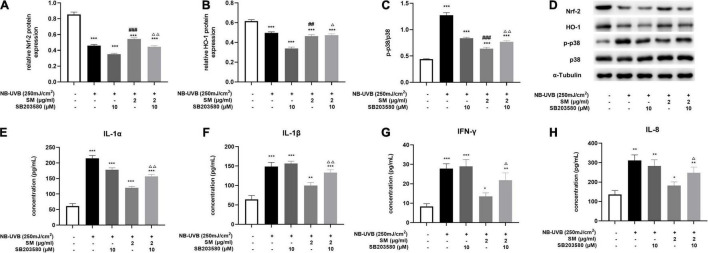
Solamargine (SM) exerting anti-inflammatory effect *via* the activation of p38 MAPK/Nrf2/HO-1 signaling pathways in HaCaT cells. After UVB irradiation of HaCaT cells, the protein level of Nrf-2 **(A)** and HO-1 **(B)** was significantly decreased, and p-p38/p38 **(C)** expression was significantly increased. This effect can be reversed by SM. The expression of Nrf-2 and HO-1 proteins in HaCaT cells was significantly decreased and p-p38/p38 expression was significantly increased in the UVB + SM + p38 inhibitor (SB203580) group compared with the UVB + SM group. After the addition of the p38 inhibitor, IL-1α **(D)** Western blot assay of Nrf-2, HO-1, p-p38 and p38 after UVB exposure with/without SM or SB203580 treatment. **(E)**, IL-1β **(F)**, IL-8 **(G)**, and IFN-γ **(H)** in the cell supernatant were significantly increased. The data represent the mean ± S.D. from three independent experiments. *P < 0.05, **P < 0.01, ***P < 0.001 (vs. non-treated group); ^Δ^*P* < 0.05, ^ΔΔ^ *P* < 0.01 (vs. NB-UVB irradiated group); ^##^*P* < 0.01, ^###^*P* < 0.001 (vs. NB-UVB irradiated and SM treated group).

The MAPK signaling pathway is an ancient classical signaling pathway closely related to inflammatory response, and p38 MAPK/Nrf2/HO-1 pathway is also involved in mediating the oxidative stress response. After UVB irradiation of HaCaT cells, compared with the negative control group, Nrf-2, HO-1 protein expression was significantly decreased, and p-p38/p38 expression was significantly increased. The expression of Nrf-2 and HO-1 proteins in HaCaT cells was significantly higher and p-p38/p38 expression was significantly lower in the UVB + SM group compared with the UVB group, indicating SM induced the activation of the Nrf-2/HO-1 pathway to attenuate oxidative stress and inflammation possibly through inhibiting p38 phosphorylation. The expression of Nrf-2 and HO-1 proteins in HaCaT cells was significantly decreased, and p-p38/p38 expression was significantly increased in the UVB + SM + p38 inhibitor (SB203580) group compared with the UVB + SM group (Figures 4A–D), indicating SM could activate the p38 MAPK/Nrf2/HO-1 signaling pathway in HaCaT cells after UVB irradiation. SB203580 is a p38 inhibitor acting primarily to block the catalytic activity of p38 MAPK by the inhibition of the activation of MAPKAP K2, a specific physiological substrate of p38 MAPK. SB203580 specifically inhibits the activity of p38 MAPK but not its activation by upstream MAPKK (30).

To verify that SM down-regulates the expression of pro-inflammatory cytokines through the p38 MAPK/Nrf2/HO-1 signaling pathway in HaCaT cells, we compared the UVB + SM + p38 inhibitor (SB203580) group with the UVB + SM group. The results showed that after the addition of the p38 inhibitor, IL-1α, IL-1β, IL-8, and IFN-γ in the cell supernatant were significantly increased as shown in Figures 4E–H, suggesting that SM inhibited the expression of pro-inflammatory cytokines through the p38 MAPK/Nrf2/HO-1 signaling pathway in HaCaT cells thereby exerting an anti-inflammatory effect.

## Discussion

Solamargine (SM), a novel natural extract from *Solanum undatum*, was previously reported as a mild anti-tumor agent for precancerous cutaneous lesions like solar keratosis. Previous researchers revealed anti-inflammatory and anti-oxidant activity of SM in skin tumor cell lines, which led to our interest in its possible therapeutic effect on PIH. This study further examined its bioactivity in human keratinocytes and melanocytes and found SM could alleviate UVB-induced inflammation and melanogenesis, making it a promising choice for PIH.

The pathogenesis of PIH relied on the activation of the epidermal pigmentary unit by primary inflammation. The indirect paracrine effect from keratinocytes and other cell types played a major role in accelerating melanin synthesis and promoting melanosome transfer. Different from previous studies on melanocytes, we focused on keratinocytes, which build up the microenvironment of melanocytes, and chose UVB irradiation as the trigger of PIH. Results showed that 2 μg/ml of SM could significantly reduce the mRNA and protein expression of inflammatory cytokines IL-1α, IL-1β, IL-8, and IFN-γ in HaCaT cells upon UVB irradiation, which was consistent with the results of classical anti-inflammatory drug dexamethasone. Besides, we also checked the changes in paracrine melanogenic factors ET-1, α-MSH, and bFGF excreted by keratinocytes upon UVB-irradiation and found similar alleviating effects of SM comparable to classical whitening ingredient arbutin. These results indicated potent anti-inflammatory effects and inhibitory effects on the paracrine melanogenic pathway, making it a possible candidate for PIH therapy from the keratinocyte’s side.

The MAPK signaling pathway is an ancient classical pathway that plays cell division, proliferation, migration, survival, apoptosis, and differentiation. MAP kinase family members, especially p38 and JNK, played crucial regulatory roles in the inflammation processes. Recently, p38 MAPK/Nrf2/HO-1 signaling pathway has become very popular as a protective pathway in response to oxidative stress (31, 32). Studies found that Asteraceae plant Dioscorea (33) alleviated inflammatory cytokines IL-1β and TNF-α *via* the p38 MAPK/Nrf2/HO-1 pathway. Another natural extract Shikonin (34) also exerted anti-inflammatory and antioxidant effects through p38 MAPK/Nrf2/HO-1 pathway by reduction of TNF-α, IL-6, IL-1β, and IFN-γ as well as activation of SOD and GSH.

Our results indicated SM could also inhibit UVB-induced p38 phosphorylation in keratinocytes. Moreover, SM inhibition of UVB-induced cytokines: IL-1α, IL-1β, IL-8, and IFN-γ was reversed by p38 MAPK inhibitor SB203580. Previous studies have proved SM exerted anti-inflammatory effects by inhibiting the NF-κB/p65 pathway and expression of COX-2 (14). Our research added that SM could also downregulate inflammatory cytokines like IL-1α, IL-1β, IL-8, and IFN-γ by inhibiting the p38 MAPK pathway. Oxidative stress is a major contributive factor in UVB-induced cutaneous inflammation. Our study found that the expression of Nrf-2 and HO-1 was significantly decreased upon UVB irradiation. Meanwhile, SM treatment significantly upregulated the expression of Nrf-2 and HO-1, consistent with its previously reported antioxidant effect, where SM reduced ROS release and downregulated the activity of i-NOS (16). Similarly, SM upregulation of Nrf-2 and HO-1 was reversed by p38 MAPK inhibitor SB203580, indicating SM might exert anti-oxidant activity through the p38 MAPK/Nrf2/HO-1 signaling pathway. Future studies on the change of ROS level in UVB-irradiated keratinocytes and melanocytes upon SM treatment could further confirm SM’s antioxidant activity.

After examination of SM’s anti-inflammatory activity and its molecular basis in keratinocytes, we then evaluated whether SM could directly interfere with melanin synthesis in UVB-irradiated melanocytes. We found that 2 μg/ml SM could significantly decrease UVB-induced melanin production in HEM cells comparable to arbutin. More specifically, SM inhibited UVB-induced upregulation of key players in melanogenesis in HEM cells: TYR, TRP-1/2, and MITF, and downregulated UVB-induced tyrosinase activation. These results indicated SM could effectively alleviate UVB-induced hyperpigmentation by suppressing the expression and activity of critical modulators in melanin synthesis in melanocytes. Korean scholars found that purple grape root extract birchic acid inhibited melanin synthesis by modulating MEK/ERK and PI3K/Akt pathways to suppress CREB, MITF, and TRP-1/2 expression in B16F10 melanoma cells (27). Centipede extract Jineol inhibited MITF and TYR through p38 and ERK pathways and downstream TRP-1/2 mRNA and protein expression to inhibit melanin synthesis (28). Whether SM inhibited melanogenesis in HEM through the same pathway is of great interest and would be a promising direction for future SM research. Notably, we used NB-UVB lamp (311 nm) rather than broad-band UVB (290–320 nm) as the irradiation source in our study and found 250 mJ/cm^2^ as the appropriate dose for melanocyte pigmentation study. Future researches might adjust the dose according to different light sources and cell lines.

Our study explored the bioactivity of SM in keratinocytes and melanocytes, and examined its anti-inflammatory effect and anti-melanogenic effect of both paracrine way and direct inhibition of melanin synthesis, making it a promising agent targeting both inflammation and hyperpigmentation for early intervention for PIH. Besides, we found SM exerted anti-inflammatory activity *via* the p38 MAPK/Nrf2/HO-1 signaling pathway, which replenished the existing molecular mechanism of SM. Further investigation of SM bioactivity could be conducted through a co-culture trans well assay or 3D skin model to provide a more direct and accurate examination of the paracrine effect and interaction between keratinocytes and melanocytes.

## Conclusion

Our study characterized the anti-inflammatory and anti-melanogenic effects of SM on UVB-irradiated keratinocytes and melanocytes model comparable to dexamethasone and arbutin, respectively. SM alleviated UVB-induced inflammation in keratinocytes *via* p38 MAPK/Nrf2/HO-1 pathway and inhibited UVB-induced melanin synthesis through downregulation of critical modulators of melanogenesis. In conclusion, SM could become a promising therapeutic agent for PIH targeting both inflammatory and pigmentary phases. Future clinical investigations could further elucidate its efficacy and safety in PIH.

## Data Availability Statement

The raw data supporting the conclusions of this article will be made available by the authors, without undue reservation.

## Author Contributions

LX: conceptualization and funding acquisition. JZ: methodology. ZL: formal analysis and investigation. YD: writing – original draft preparation. QW, MJ, and CZ: writing – review and editing. H-MS and C-SL: resources. LX, H-MS, and C-SL: supervision. All authors contributed to the article and approved the submitted version.

## Conflict of Interest

The authors declare that the research was conducted in the absence of any commercial or financial relationships that could be construed as a potential conflict of interest.

## Publisher’s Note

All claims expressed in this article are solely those of the authors and do not necessarily represent those of their affiliated organizations, or those of the publisher, the editors and the reviewers. Any product that may be evaluated in this article, or claim that may be made by its manufacturer, is not guaranteed or endorsed by the publisher.
